# Atypical metastases from prostate cancer detected on 68Ga-PSMA PET/CT: a case series

**DOI:** 10.1590/S1677-5538.IBJU.2020.0069

**Published:** 2020-11-18

**Authors:** Lilian Yuri Itaya Yamaga, Marcelo Livorsi da Cunha

**Affiliations:** 1 Hospital Israelita Albert Einstein Departamento de Imagem São PauloSP Brasil Departamento de Imagem, Hospital Israelita Albert Einstein, São Paulo, SP, Brasil

## CASES PRESENTATION

Prostate cancer (PCa) is one of the most frequent malignant tumors in men worldwide. The primary treatment of localized disease consists of radical prostatectomy and radiation therapy. Unfortunately, tumor recurrence after initial treatment is not uncommon and is suspected by the rise in prostate––specific antigen (PSA) levels. Distinguishing between a local recurrence and distant metastases is critical to define an effective therapy (1).

The most common pattern of tumor spread involves abdominopelvic lymph nodes and the bones followed by the lung, liver, pleura, supradiaphragmatic lymph nodes and adrenal glands. Rarer metastatic sites may be observed in nearly any organ such as the brain, breast, diaphragm, gastrointestinal tract, skin, heart, penis and testicle (2–5).

Traditionally, PCa recurrence is investigated by prostate imaging such as computed tomography, magnetic resonance and bone scintigraphy. However, this approach has limited sensitivity particularly at low PSA levels (1).

The recently introduced positron emission tomography/computed tomography (PET/CT) with the new tracer 68Ga-labeled prostate specific membrane antigen ligand (PSMA) has revolutionized the evaluation of patients with PCa recurrence. PSMA is a transmembrane enzyme which is significantly overexpressed in the majority of prostatic adenocarcinomas. PSMA expression rises with increasing tumor dedifferentiation, metastatic and hormone-refractory cancers. PET/CT with 68Ga-PSMA provides a high detection rate in the evaluation of local recurrence or metastatic disease with the summary sensitivity and specificity of 86% on per-patient analysis. PET/CT positive results increase with PSA level and shorter PSA doubling time (6–8).

However, to date, a few investigations have described rare sites of metastases from PCa detected by PET/CT with 68Ga-PSMA (9, 10).

**Figure 1:**
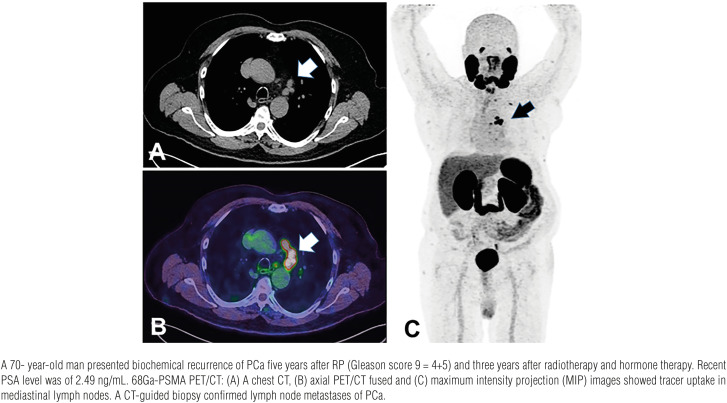
Mediastinal lymph nodes metastases.

**Figure 2:**
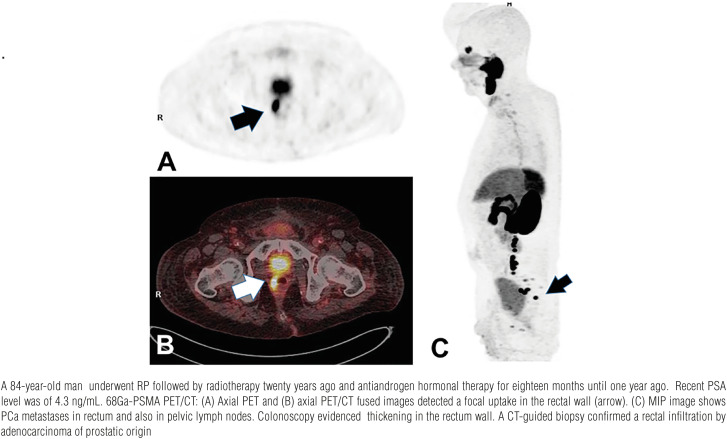
Rectal metastasis.

**Figure 3:**
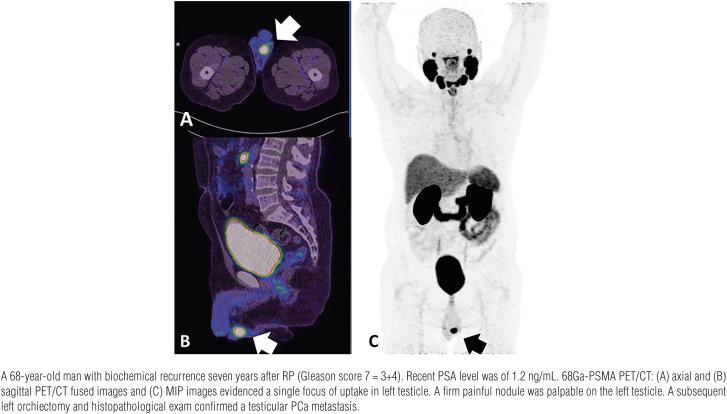
Testicular metastasis.

**Figure 4:**
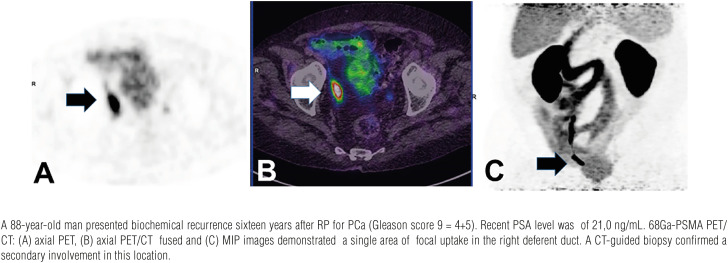
Deferent duct metastasis.

**Figure 5:**
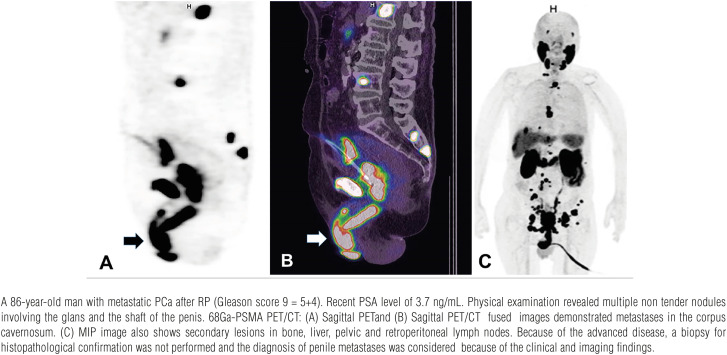
Penile metastases.

**Figure 6:**
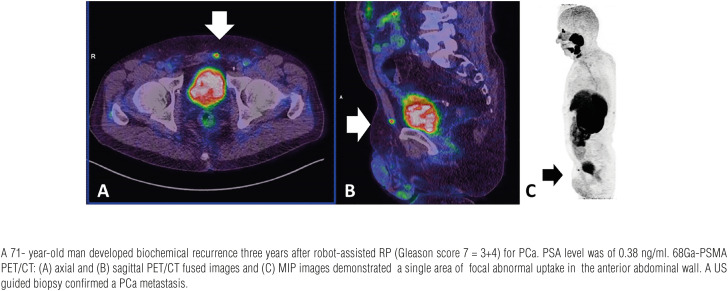
Abdominal wall metastasis.

We report a case series in which atypical sites of PCa metastases in mediastinal lymph nodes (Figure-1), rectum (Figure-2), testis (Figure-3), deferent duct (Figure-4), penis (Figure-5) and abdominal wall (Figure 6) were detected by PET/CT with 68Ga-PSMA. Of note, a single atypical metastasis was detected by PET/CT in each of three of the six reported cases (Figures 3, 4 and 6). In this context, PET/CT with 68Ga-PSMA had an effective role not only in the detection of the PCa metastasis but also in the treatment planning.

## CONCLUSIONS

PET-CT with 68Ga-PSMA allows the detection of PCa metastases, including the rarer sites, proving its diagnostic value in the evaluation of the extent of the disease in patients with recurrence.
